# Case Report: IgG4-related kidney disease complicated by interstitial pneumonia

**DOI:** 10.12688/f1000research.131818.2

**Published:** 2023-09-27

**Authors:** Akira Mima, Rina Lee, Ami Murakami, Hidemasa Gotoda, Ryosuke Akai, Shinji Lee

**Affiliations:** 1 Department of Nephrology, Osaka Medical and Pharmaceutical University, Takatsuki, Japan

**Keywords:** IgG4-related kidney disease, tubulointerstitial nephritis, interstitial pneumonia, prednisolone

## Abstract

Immunoglobulin G4 (IgG4)-related disease is a systemic inflammatory disorder characterized by tubulointerstitial nephritis with IgG4-positive plasma cell infiltration. We report the case of an 84-year-old male who presented with a history of dyspnea on exertion and cough. The lymph nodes were palpated in the axilla. Urinalysis revealed mild proteinuria and increased levels of NAG and β2-microglobulin. Blood tests showed hyperglobulinemia with a marked elevation of serum IgG4 levels. Chest computed tomography showed bilateral ground-glass and reticular opacities in the lower and peripheral portions of the lungs. Ga-67 scintigraphy showed kidney uptake.

The patient was diagnosed with IgG4-related kidney disease based on the renal pathology indicative of typical tubulointerstitial nephritis with extensive IgG4-positive plasma cell infiltration. The patient was treated with prednisolone and showed a prompt response in his clinical condition. The patient achieved normalization of serum IgG4 levels 6 months after the initiation of treatment. Although IgG4-related disease is thought to be potentially associated with organ fibrosis, there are few reports on combination of interstitial pneumonia and IgG4-related kidney disease. Our case report presents a possible pattern of IgG4-related disease.

## Introduction

Immunoglobulin G4 (IgG4)-related disease is a systemic inflammatory disease characterized by extensive lymphoplasmacytic infiltration of IgG4-positive cells in various organs. ^1^^–^^3^ Renal involvement has been reported in approximately 10% of patients with IgG4-related disease. ^4^ Respiratory organ lesions have also been recognized; however, their rate is low and they are often only detected during close examination. ^5^ Clinical symptoms vary depending on the organs affected by IgG4-related disease; however, they are usually mild. Thus, IgG4-related disease should be diagnosed based on a combination of clinical, serological, and radiological findings, and pathological features. ^6^ There have only been a few reports on the coexistence of interstitial pneumonia and IgG4-related kidney disease. ^7^ In this report, we describe the case of a patient who presented with interstitial pneumonia and was diagnosed with IgG4-tubulointerstitial nephritis and IgG4-related kidney disease. Treatment with prednisolone was initiated soon after diagnosis and the patient responded well.

## Case presentation

**Figure 1.**
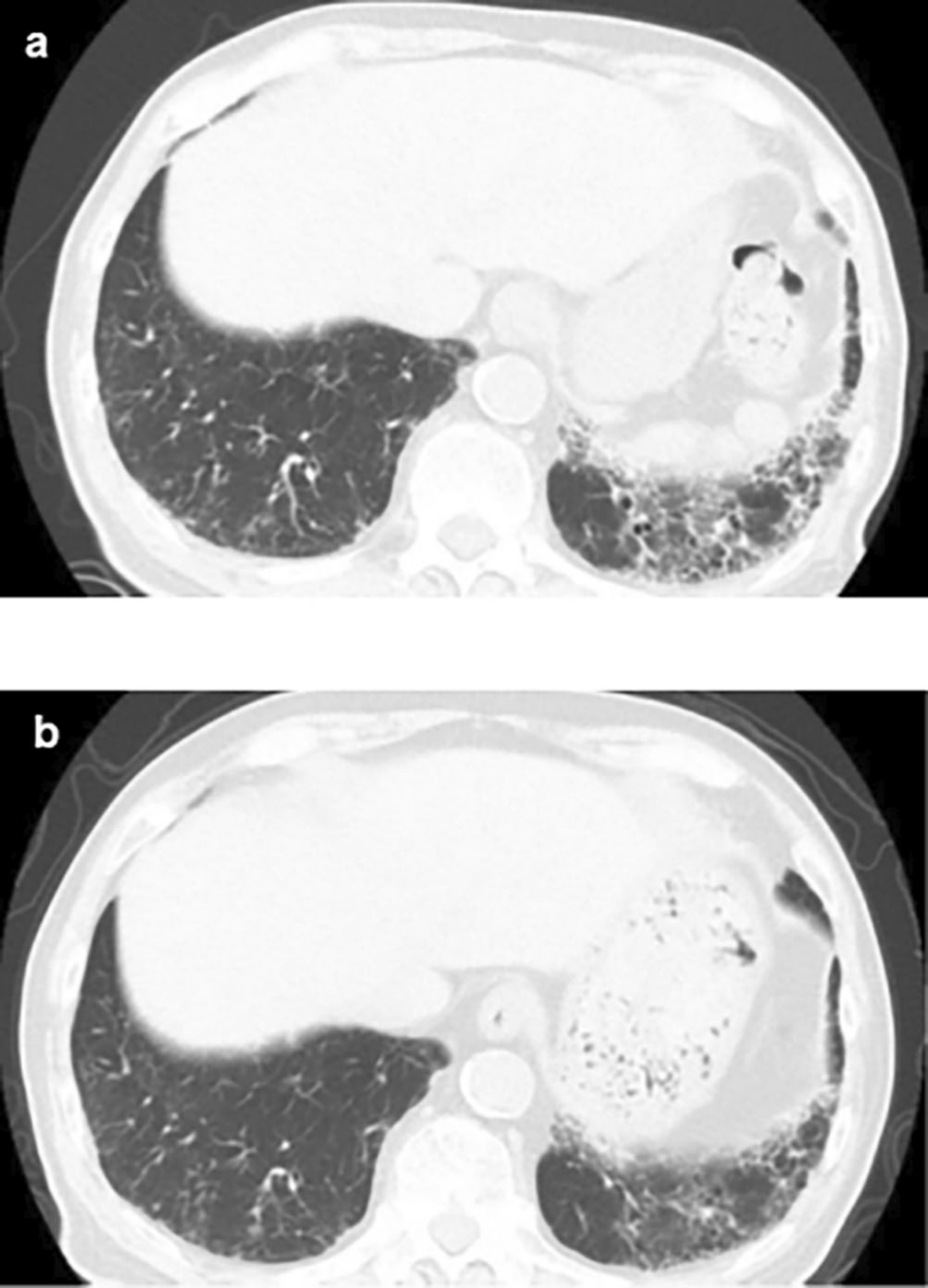
Computed tomography findings. (a) Plain chest CT showing the reticular pattern and bronchial dilatation in the bilateral lower lung fields before therapy; (b) After therapy, the interstitial pattern nearly disappeared from the bilateral lung fields. CT: computed tomography.

**Figure 2.**
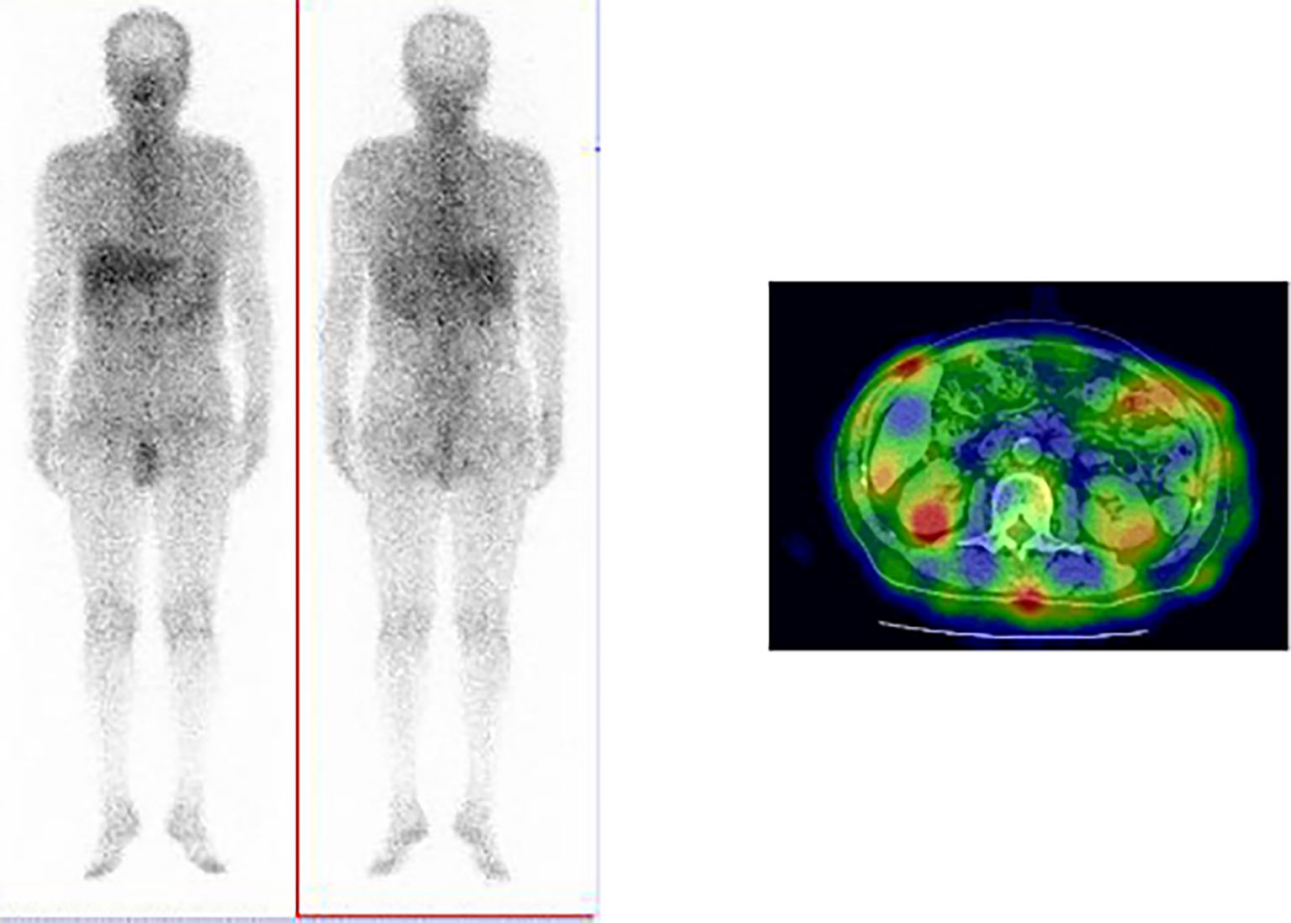
Ga-67 scintigraphy findings. Uptake by kidney revealed using Ga-67 scintigraphy.

**Figure 3.**
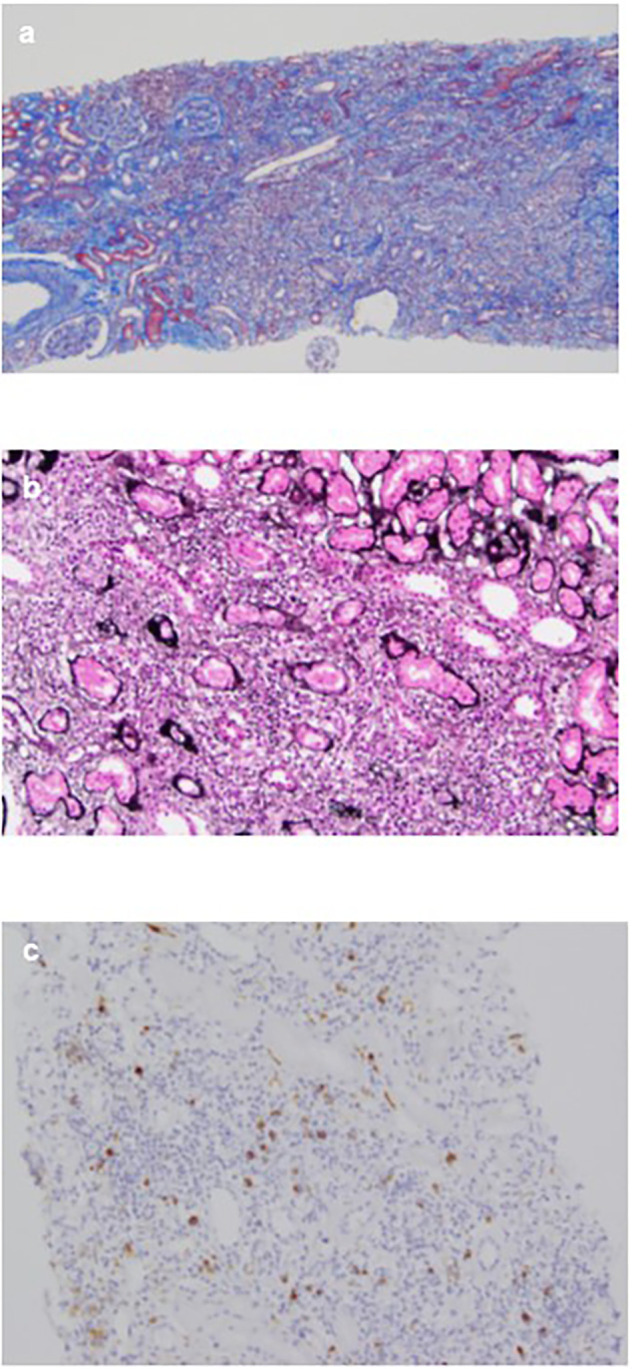
Microscopy findings of the renal biopsy. (a) The interstitium shows mononuclear cell infiltration and interstitial fibrosis with tubular atrophy with Masson’s trichrome stain (×40); (b) PAM stain in the interstitium shows bird’s eye pattern of fibrosis (×200); (c) Immunohistochemical analysis for IgG4 shows dominant IgG4+ plasma cell filtration, with an IgG4+/IgG+ ratio of >40% (×100).

**Figure 4.**
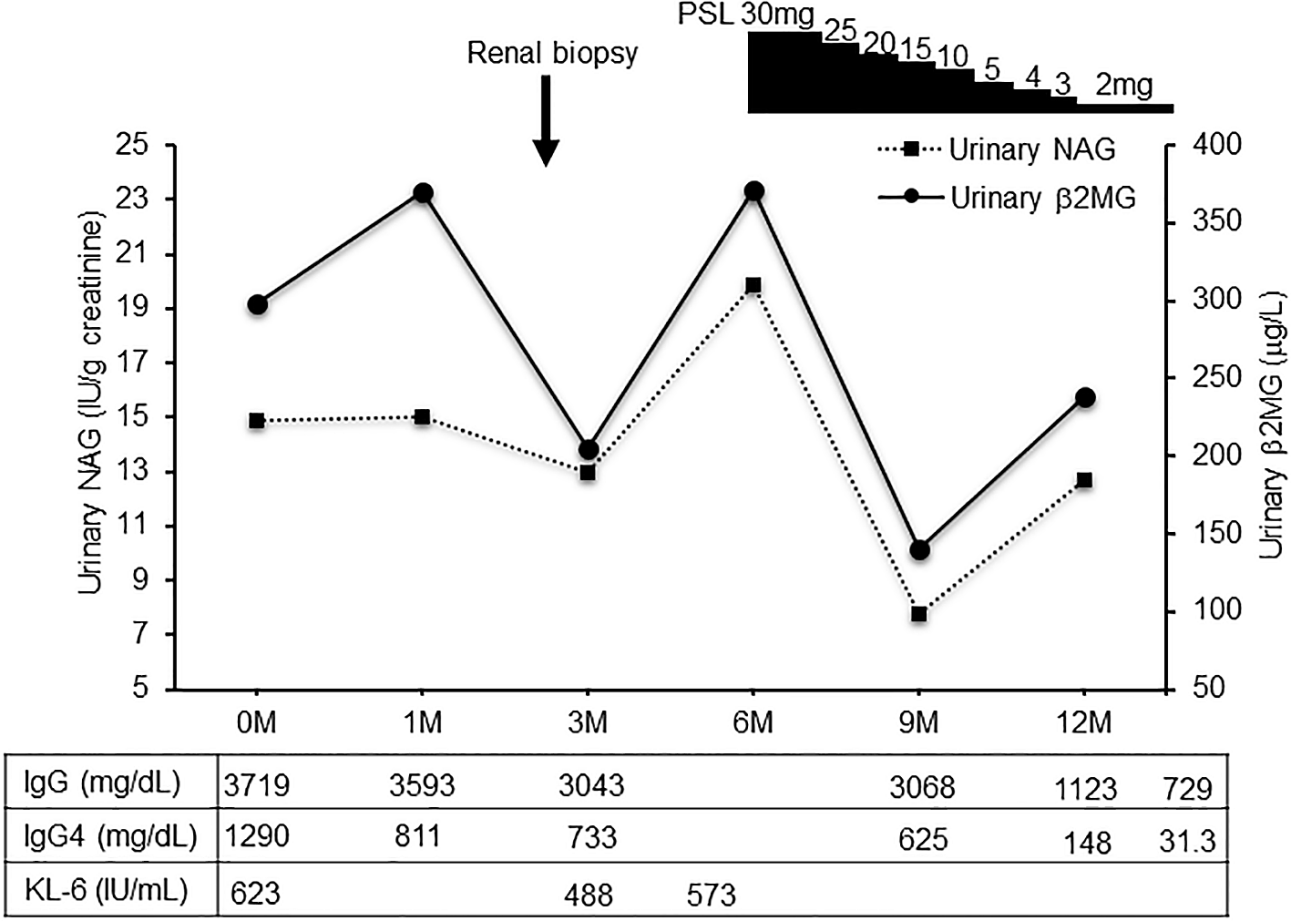
The clinical course of the patient. Clinical course treatment was initiated by administering prednisolone at 30mg/day, followed by prompt alleviation of the dyspnea on exertion and cough. Urinary NAG and β2 MG, and serum IgG4 were significantly decreased after prednisolone treatment. NAG, N-acetyl-β-D-glucosaminidase; β2 MG, β2 microglobulin; PSL, prednisolone.

An 84-year-old unemployed Japanese man presenting with dyspnea on exertion and cough was referred to our hospital. The patient had a history of hypertension and hypothyroidism, but an unremarkable family history of any related pathology. Examination results of the patient were found to be negative for arthralgia, skin rash, macrohematuria, and hemoptysis. Upon admission, body temperature was recorded to be 36.5 °C; blood pressure, 128/72 mmHg; heart rate, 72 bpm; oxygen saturation, 98%; weight, 60.5 kg; height, 164.9 cm; and BMI, 21.1 kg/m ^2^. Initial laboratory and diagnostic workup revealed blood urea nitrogen 18 mg/dL (normal range: 8-20 mg/dL), creatinine 0.98 mg/dL (normal range: 0.65-1.07 mg/dL), estimated glomerular filtration rate 56 mL/min/1.73 m ^2^ (normal range: ≥ 60 mL/min/1.73 m ^2^), calcium 9.4 mg/dL (normal range: 8.8-10.4 mg/dL), total protein 9.1 g/dL (normal range: 6.5-8.0 g/dL), albumin 3.6 g/dL (normal range: 3.9-4.9 g/dL), alkaline phosphatase 60 IU/L (normal range: 50-350 IU/L), aspartate transaminase 27 IU/L (normal range: 7-38 IU/L), alanine transaminase 11 IU/L (normal range: 4-44 IU/L), total cholesterol 182 mg/dL (normal range: 120-220 mg/dL), triglyceride 175 mg/dL (normal range: 50-149 mg/dL), WBC 11.5×10 ^3^/μL (normal range: 3.1-8.4×10 ^3^/μL), RBC 3.97×10 ^6^/μL (normal range: 4.2-5.7×10 ^6^/μL), hemoglobin 12.5 g/dL (normal range: 14-18 g/dL), platelets 200×10 ^3^/μL (normal range: 150-330×10 ^3^/μL), eosinophils 3% (normal range: 0-5%), and urinalysis negative for protein, RBC, and cell casts. Urinary N-acetyl-beta-D-glucosaminidase (NAG)/creatinine 17.1 IU/gCr (normal range: 1.6-5.8 IU/gCr) and urinary β2 microglobulin 371 mg/L (normal range: ≤289 mg/L). Antineutrophil cytoplasmic antibodies screening was found to be negative, with low C3=68 mg/dL, low C4=7.4 mg/dL, and negative for anti-SSA, anti-SSB, anti-RNP, Scl-70, anti-Sm, and ds-DNA antibodies. Antinuclear antibodies (ANA) antibodies were found to be ×1280. Hyperglobulinemia was determined with an IgG level of 3719 mg/dL (normal range: 870-1700 mg/dL) and an IgG4 level of 1290 mg/dL (normal range: 4-108 mg/dL). Rheumatoid factor (≤15 IU/mL) was elevated at 24 IU/mL, and KL-6 (<500 IU/mL) was significantly increased at 623 IU/mL. Computerized tomography (CT) revealed bilateral ground-glass and reticular opacities predominantly in the lower and peripheral portions of the lungs ( Figure 1a). Furthermore, bronchial wall thickening, and enlarged cervical, mediastinal, and axillary lymph nodes were identified. However, renal, pancreatic, or salivary gland inflammation was not observed. Ga-67 scintigraphy revealed accumulation in the kidneys ( Figure 2). Consequently, the patient was diagnosed with IgG4-related kidney disease based on the renal pathology with massive tubulointerstitial nephritis, characteristic fibrosis (bird’s eye pattern) ( Figure 3a and b), and IgG4-positive cell infiltration, wherein the number of IgG4 positive plasma cells was >10/hpf, and IgG4/IgG ratio was 61.9% ( Figure 3c). Deposition of globulin or complement, and evidence of glomerular sclerosis were not observed in the glomeruli. Treatment was initiated by administering oral prednisolone at 30 mg/day for one month, followed by prompt alleviation of cough and dyspnea on exertion. Furthermore, oxygen saturation was quickly restored to 100%. With a subsequent decrease (after one month prednisolone treatment) in urinary NAG and β2 microglobulin and IgG4 levels, prednisolone was decreased from 2.5 to 5 mg every 2 to 4 weeks for 4 weeks. One year after the initiation of treatment, the patient achieved normalization of serum IgG4 levels, and chest CT revealed the interstitial pattern was found to have nearly disappeared ( Figure 1b). Figure 4 shows the clinical course of this patient.

## Discussion

IgG4-related diseases should be differentiated from diseases caused by excessive inflammatory cytokine production, such as interleukin-6 (IL-6)-produced Castleman’s disease and autoimmune disorders. ^8^ Usually, IgG4-related diseases are observed in middle-aged men. Most patients with this disease present with associated extrarenal lesions, such as in the salivary glands, lymph nodes, and pancreas. ^9^ Therefore, the clinical manifestations of the disease vary according to the organ involved. The most characteristic features of IgG4-related kidney disease are presence of IgG4 positive plasma cell-rich tubulointerstitial nephritis and fibrosis, which are sometimes concurrent with glomerular lesions. ^10^^,^^11^

Similar to the present case, approximately 30% of the patients diagnosed with IgG4-related kidney disease do not show any abnormality on a CT scan. In contrast, previous reports indicated that approximately 70% of patients with IgG4-related kidney disease show some kind of abnormality. ^9^^,^^12^ The discrepancy between these findings can be owing to the presence or absence of contrast-enhanced CT, which is necessary within renal function tolerance. Ga-67 scintigraphy and FDG-PET have been reported to be useful for its diagnosis. However, contradictory to the present case, positive accumulation in the kidney during Ga-67 scintigraphy has been reported to be only 11%. ^9^ Another useful method of analysis for IgG4-related kidney disease is T2-weighted and diffusion-weighted MR imaging. ^13^

CT imaging findings of the lungs in IgG4-related diseases have been classified into four types: ground-glass opacity, diffuse reticular, bronchial wall thickening, and nodular patterns. ^14^ The ground-glass opacity and diffuse reticular patterns potentially suggest lymphocyte and plasma cell infiltration of the alveoli and interstitium. Bronchial wall thickening pattern indicates plasma cells infiltration of the bronchial vascular bundles, alveoli, and interstitium. Finally, nodular sclerosing inflammation of the bronchial glands was prominently observed to be associated with the nodular pattern. ^14^ However, in some lymphoproliferative diseases other than IgG4-related diseases, similar findings are seen on chest imaging or histopathology, and it is difficult to differentiate them from respiratory involvement of IgG4-related diseases, suggesting that respiratory involvement of IgG4-related diseases should be diagnosed comprehensively from the clinical picture, imaging findings and pathological aspects. Furthemore, relatively good response to corticosteroid has been reported for the treatment of IgG4-related lung lesions. ^15^ Although we did not perform a lung biopsy in the present case, the CT findings of diffuse reticular and bronchial wall thickening confirmed pulmonary involvement in IgG4-related diseases.

Inflammatory cytokines, such as IL-5 and tumor necrosis factor (TNF)-α, reportedly correlate with IgG4-related diseases. ^16^^,^^17^ Furthermore, our previous reports also indicate that the inflammatory cytokines play a potentially pivotal role in the development of renal fibrosis. ^18^ T helper cells type 2 (Th2) and regulatory T cells (Tregs) have been reported to be involved in IgG4-related tubulointerstitial fibrosis. ^19^ Treg cells can increase the regulatory cytokines, such as transforming growth factor-β (TGF-β), which has also been identified in renal fibrosis signaling. ^19^^,^^20^ However, many aspects of this disease, especially complications caused by interstitial pneumonia, remain unclear with limited reports on this disease type; hence, further studies are warranted.

The optimal treatment for IgG4-related kidney disease has not been established; however, most patients, including the one in the present case, respond to prednisolone. Saeki *et al.* showed that the induction of prednisolone led to rapid improvement in renal function and serological abnormalities 1 month after the initiation of therapy, and that maintenance therapy with low-dose prednisolone successfully resulted in long-term suppression of disease activity in 43 Japanese patients with IgG4-related kidney disease. ^9^

Similar to the present case, even in patients without diagnosis of renal localized IgG4-related disease, favorable outcomes can be achieved with the same treatment modalities as those employed for the patients diagnosed with the disease.

IgG4-related kidney disease is an emerging disease with recently identified pathological features. Generally, patients respond rapidly to prednisolone and present a relatively favorable prognosis. The present case of IgG4-related kidney disease associated with interstitial pneumonia provides a novel rationale for its pathophysiology.

## Consent

Written informed consent was obtained from the patient for publication of the details of their medical case and any accompanying images.

The study protocol for the patient record review was reviewed and the need for approval was waived by Institutional Review Board of Osaka Medical and Pharmaceutical University, as a retrospective review of patient data did not require ethical approval in accordance with local guidelines.

## Data Availability

All data underlying the results are available as part of the article and no additional source data are required.
