# Supplementary material for: Case Report: IgG4-related kidney disease complicated by interstitial pneumonia
Source: F1000Res. 2023 Sep 27;12:1045. Originally published 2023 Aug 29. [Version 2] doi: 10.12688/f1000research.131818.2 (PMC10511842; doi:10.12688/f1000research.131818.2)
Supplement: Peer review history

# Reviewer report: Reviewer response for version 2

**DOI:** 10.5256/f1000research.156395.r210217

**Published:** 2023-09-28

**Authors:** Tomohito Gohda

**Affiliations:** 1 Department of Nephrology, Faculty of Medicine, Juntendo University, Tokyo, Japan

The authors have revised appropriately.

Are enough details provided of any physical examination and diagnostic tests, treatment given and outcomes?

Yes

Is the case presented with sufficient detail to be useful for other practitioners?

Yes

Is sufficient discussion included of the importance of the findings and their relevance to future understanding of disease processes, diagnosis or treatment?

Partly

Is the background of the case’s history and progression described in sufficient detail?

Yes

Reviewer Expertise:

diabetic kidney disease

I confirm that I have read this submission and believe that I have an appropriate level of expertise to confirm that it is of an acceptable scientific standard.

# Reviewer report: Reviewer response for version 1

**DOI:** 10.5256/f1000research.144701.r203748

**Published:** 2023-09-20

**Authors:** Seiji Kishi

**Affiliations:** 1 Department of Nephrology and Hypertension, Kawasaki Medical School, Kurashiki, Japan

This case report presents an IgG4-related disease complicated by pulmonary involvement. The association with pulmonary lesions is discussed as a novel finding.

 Please allow me to ask the following questions to improve the paper and make it more meaningful to the reader:

1. Are there any other findings besides imaging that can be evaluated in relation to steroid therapy? Physical examination, walking distance, oxygen saturation, pulmonary function tests, and even other blood tests would be acceptable.
2. Can you discuss more about IgG4-related diseases and pulmonary involvement? For example, the classification and frequency of interstitial pneumonia, the prediction of steroid response, and whether the findings are predictive of recovery of renal function.

Are enough details provided of any physical examination and diagnostic tests, treatment given and outcomes?

Yes

Is the case presented with sufficient detail to be useful for other practitioners?

Yes

Is sufficient discussion included of the importance of the findings and their relevance to future understanding of disease processes, diagnosis or treatment?

Partly

Is the background of the case’s history and progression described in sufficient detail?

Yes

Reviewer Expertise:

kidney disease, acute kidney injury, chronic kidney disease

I confirm that I have read this submission and believe that I have an appropriate level of expertise to confirm that it is of an acceptable scientific standard, however I have significant reservations, as outlined above.

# Reviewer report: Reviewer response for version 1

**DOI:** 10.5256/f1000research.144701.r203747

**Published:** 2023-09-11

**Authors:** Tomohito Gohda

**Affiliations:** 1 Department of Nephrology, Faculty of Medicine, Juntendo University, Tokyo, Japan

This case report is well written. However, I suggest the authors to respond to the following comments:

1. The quality of microscopy findings of renal biopsy seems to be poor.
2. Please describe in more detail how to differentiate normal IP from IgG4-related lung disease in the absence of lung biopsy findings.
3. Please describe any differences from previous reports in cases of IgG4-related renal and respiratory disease.

Are enough details provided of any physical examination and diagnostic tests, treatment given and outcomes?

Yes

Is the case presented with sufficient detail to be useful for other practitioners?

Yes

Is sufficient discussion included of the importance of the findings and their relevance to future understanding of disease processes, diagnosis or treatment?

Partly

Is the background of the case’s history and progression described in sufficient detail?

Yes

Reviewer Expertise:

diabetic kidney disease

I confirm that I have read this submission and believe that I have an appropriate level of expertise to confirm that it is of an acceptable scientific standard, however I have significant reservations, as outlined above.

# Reviewer report: Reviewer response for version 1

**DOI:** 10.5256/f1000research.144701.r203749

**Published:** 2023-09-11

**Authors:** Masaru Matsui

**Affiliations:** 1 Department of Nephrology, Nara Medical University, Nara, Japan

This case report focuses on the topic that interstitial pulmonary diseases are a rare complication of IgG4-related diseases. As the authors acknowledge, lung biopsy was required for showing IgG4 cells infiltration in interstitium of lungs, and therefore the pathological association between lung and kidney may not be evident. This reviewer was curious about changes in KL-6 after steroid treatment.

Are enough details provided of any physical examination and diagnostic tests, treatment given and outcomes?

Yes

Is the case presented with sufficient detail to be useful for other practitioners?

Yes

Is sufficient discussion included of the importance of the findings and their relevance to future understanding of disease processes, diagnosis or treatment?

Yes

Is the background of the case’s history and progression described in sufficient detail?

Yes

Reviewer Expertise:

CKD

I confirm that I have read this submission and believe that I have an appropriate level of expertise to confirm that it is of an acceptable scientific standard.
